# Hemothorax caused by vertebral exostoses

**DOI:** 10.11604/pamj.2019.33.232.17846

**Published:** 2019-07-18

**Authors:** Jamel EL Ghoul, Soued Ferjani

**Affiliations:** 1 Pulmonary Disease and Critical Care Service, Medenine Hospital, Medenine, Tunisia; 2 Department of Radiology, Medenine Hospital, Medenine, Tunisia

**Keywords:** Hemothorax, exostosis, thoracic vertebrae

## Image in medicine

Spontaneous hemothorax is a rare condition and sometimes occurs during anticoagulant therapy for venous thromboembolism. Other causes of the condition are bleeding disorders, complication of spontaneous pneumothorax and pleural malignancy. Vertebral exostosis represents an exceptional etiology of hemothorax, as a result of direct traumatic injury of the pleura or diaphragm by the intrathoracic tumour. Exostosis often occurs in hereditary multiple exostoses, but can also be isolated. We present a case of spontaneous hemothorax in a patient with vertebral exostoses and propose a new etiology of hemothorax. A 22-year-old man with no specific history elements and no previous trauma presented has been experiencing a right-sided pleuritic chest pain and respiratory discomfort for 6 days. Physical examination revealed dullness to percussion and decreased breath sounds on the right side of the chest. Chest x-ray showed right pleural effusion and diagnostic thoracocentesis revealed gross blood. Pleural effusion's hematocrit was 52%, while that of the peripheral blood was 39%. Hemothorax was diagnosed. Thoracic Computed tomography scan (CT-scan) (A and B, arrows) followed by magnetic resonance imaging (MRI) showed the presence of two vertebral exostoses arising from the right transverse processes of the 10^th^ and 11^th^ thoracic vertebrae (C and D, arrows). These, encroached on the 11^th^intercostal space. The hemothorax was managed successfully with chest-tube insertion. However, surgical resection of exostoses was indicated in our patient given the risk of loco-regional complications, and the recurrence of the hemothorax. The postoperative course was uneventful, and the patient was followed without recurrent hemothorax for two years.

**Figure 1:**
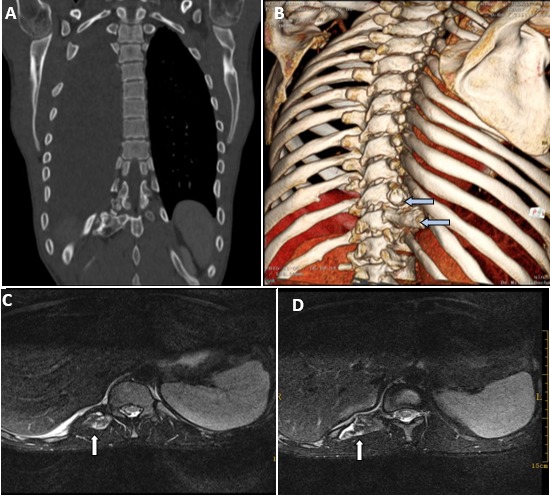
(A) computed tomography of the chest, coronal view with 3-dimension reconstruction; (B) presence of two focal bony masses arising from the right transverse processes of thoracic vertebrae 10 and 11; (C) magnetic resonance imaging of the spine, T2-weighted sequence, axial section, shows two vertebral exostoses arising from the right transverse processes of thoracic vertebrae 10 and 11; (D) measuring 20 × 12 mm and 35 × 25 mm, respectively. That of the transverse process of T11 encroaches on the 11^th^ intercostal space

